# Granuloprival cerebellar cortical degeneration in a Yorkshire Terrier and Lagotto Romagnolo dog

**DOI:** 10.1111/jvim.17091

**Published:** 2024-04-25

**Authors:** Thomas Mignan, Martí Pumarola, Simon Platt, Matthew James, Marta Pereira, Antonia Morey‐Matamalas, Alfredo Recio

**Affiliations:** ^1^ Dovecote Veterinary Hospital, 5 Delven Lane, Castle Donington Derby DE74 2LJ United Kingdom; ^2^ Department of Animal Medicine and Surgery, Veterinary Faculty Universitat Autònoma de Barcelona, Bellaterra (Cerdanyola del Vallès) Barcelona 08193 Spain; ^3^ CVS Teleneurology, 1 Owen Road Diss IP22 4ER United Kingdom; ^4^ School of Veterinary Medicine and Science, University of Nottingham Sutton Bonington, Leicestershire LE12 5RD United Kingdom; ^5^ Clínica Veterinaria Levante, Avenida de La Unión 61 San Javier, Murcia 30730 Spain

**Keywords:** abiotrophy, canine, cerebellum, degenerative, granular cell

## Abstract

Granuloprival degeneration is an uncommon form of cerebellar cortical degeneration (CCD). A 3‐month‐old Yorkshire Terrier and a 7‐month‐old Lagotto Romagnolo dog were presented with a history of progressive cerebellar dysfunction including wide‐based stance, cerebellar ataxia, intention tremors, and loss of menace response despite normal vision. Magnetic resonance imaging of the brain identified marked diffuse decrease of the cerebellum size. Euthanasia was performed in both cases because of progression of clinical signs. Histopathological examination identified marked diffuse thinning of the granular cell layer with almost complete loss of the granular cell neurons, providing a definitive diagnosis of granuloprival CCD. Granuloprival CCD should be considered as a differential diagnosis in Yorkshire Terrier and Lagotto Romagnolo dogs with post‐natal progressive clinical signs of cerebellar dysfunction.

**Abbreviations**
CCD: cerebellar cortical degeneration

## 1 INTRODUCTION

Cerebellar cortical degeneration (CCD) describes a group of diseases characterized by postnatal degeneration predominantly affecting the cerebellar cortex.^1^ Cerebellar cortical degeneration is classified according to the primary cell target, whether Purkinje neurons or the granule cell neurons of the cerebellar cortex.^1^ In the majority of dogs with CCD, the Purkinje neuron is the primary target with secondary retrograde degeneration of the granule cell neurons.^1^ Sporadic cases of selective loss of granule cell neurons with relative sparing of the Purkinje neurons, called granuloprival degeneration, are also reported.^2^, ^3^, ^4^, ^5^, ^6^, ^7^, ^8^, ^9^, ^10^, ^11^ These include reports of several puppies in a single litter being affected in the Border Collie, Labrador Retriever, Bavarian Mountain dog, and Coton de Tuléar breed, as well as single reported cases in the Australian Kelpie, Italian hound, and Lagotto Romagnolo breed.^2^, ^3^, ^4^, ^5^, ^6^, ^7^, ^8^, ^9^, ^10^, ^11^

Herein, we respectively report the first and second histopathologically confirmed cases of granuloprival CCD in the Yorkshire Terrier and Lagotto Romagnolo breeds.

## 2 CASE PRESENTATION

### 2.1 Case 1

**FIGURE 1:**
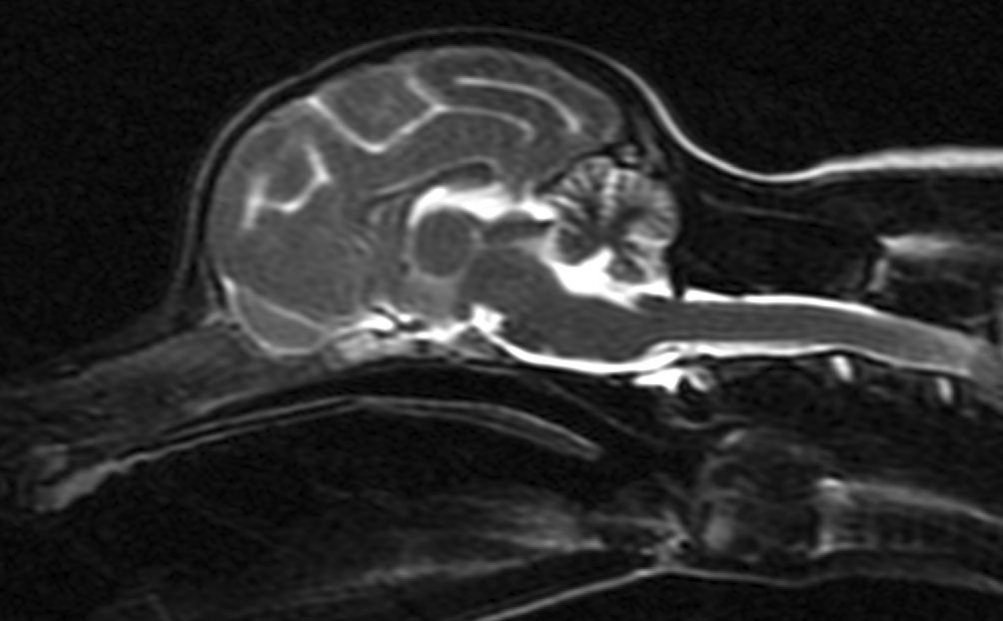
T2‐Weighted midline sagittal magnetic resonance image of the brain for Case 1.

**FIGURE 2:**
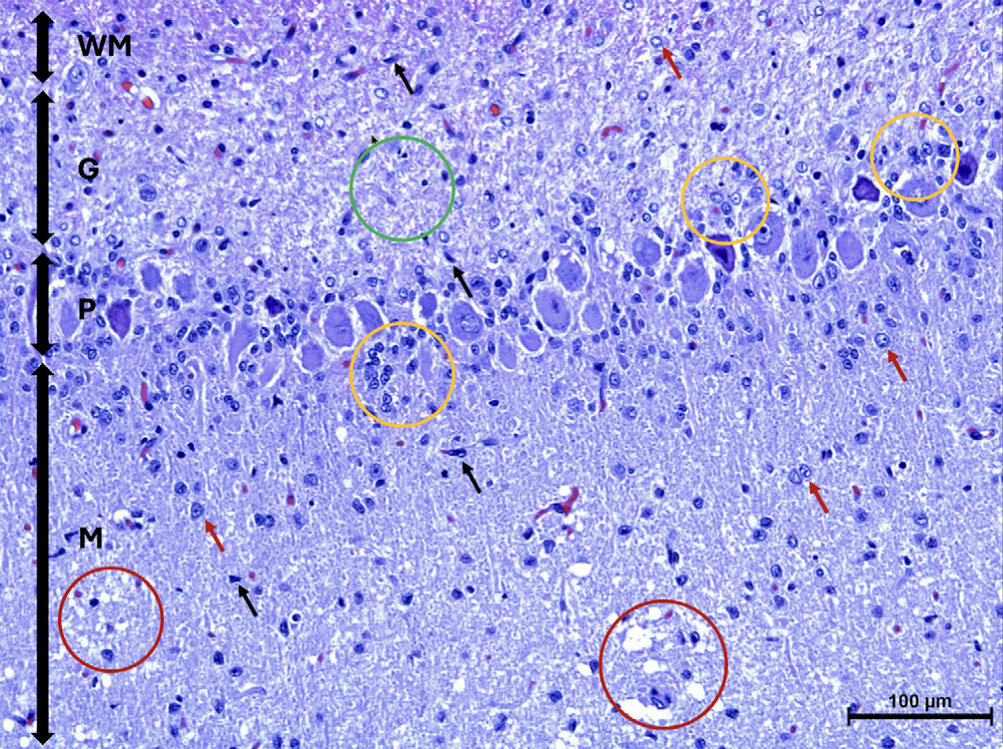
Microscopic image of the cerebellar cortex of Case 1 stained with Hematoxylin and Eosin. The granular cell layer (G) is displaying marked pallor because of hypocellularity of the granule cell neurons (green circle). There are Bergmann glial cells (yellow circles) located adjacent to the Purkinje cell layer (P). The molecular layer (M), is displaying mild astrogliosis (red arrows) including the presence of microglia (black arrows) and spongiosis (red circles). White matter (WM).

A 3‐month‐old intact male Yorkshire Terrier was presented with a history of progressive cerebellar ataxia involving all limbs and intention tremors noticed since 8 weeks of age. Eye movements were reported to be normal. All 4 littermates of the puppy were reported to be normal. General examination did not disclose any abnormality. Neurological examination identified a wide‐based stance, as well as cerebellar ataxia characterized by hypermetria involving all limbs, intention tremors of the head and neck, and bilaterally absent menace response despite normal vision. The remainder of the neurological examination was normal. Neuroanatomical localization was to the cerebellum. Differential diagnoses included a congenital malformation of the cerebellum such as Dandy Walker malformation, cerebellar hypoplasia, neosporosis, CCD, as well as a hypomyelination disorder or neuroaxonal dystrophy. Serum biochemistry and CBC were normal. Serology for *Toxoplasma gondii* and *Neospora caninum* was negative. Magnetic resonance imaging (Signa Excite 1.5 Tesla, General Electric Company, Wisconsin, United States of America) of the head was performed using a knee coil (QuadKnee Coil, General Electric Company, Wisconsin, United States of America), under general anesthesia. Magnetic resonance imaging disclosed marked diffuse decreased size of all lobes and lobules of the cerebellar vermis and hemispheres with marked widening of all cerebellar fissures, most obvious at the primary fissure, containing fluid isointense to cerebrospinal fluid on T1‐weighted, T2‐weighted, and fluid attenuation inversion recovery sequences (Figure 1). No transforaminal herniation was observed. The brainstem‐to‐cerebellum area ratio was 101% (normal, <96%) indicative of decreased cerebellum size (Figure 1).^12^ No contrast enhancement was observed. Cerebrospinal fluid was collected from the cerebellomedullary cistern under general anesthesia, and analysis, including total nucleated cell count and protein concentration, was normal. Polymerase chain reaction testing on cerebrospinal fluid for *Neospora caninum* and *Toxoplasma gondii* was negative. Owing to graded progressive deterioration of clinical signs 2 weeks after initial presentation leading to inability to ambulate and eat without assistance, the owner elected for euthanasia and consented for necropsy examination. On necropsy, all lobes and lobules of the cerebellar vermis and hemispheres were markedly decreased in size with marked thinning of the cortex upon sectioning. Microscopically, the cerebellar folia were markedly flattened. Marked diffuse thinning and hypocellularity of the granular cell layer with marked loss of granular cell neurons was observed, leading to almost complete absence of this layer with moderate gliosis (Figure 2). The Purkinje neuronal cell bodies were increased in size with accumulation of pale, granular material, causing displacement and accumulation of Nissl granules around the nuclei. This same material had accumulated in the molecular layer, and was associated with moderate gliosis composed mainly of astrocytes with some Bergmann glial cells. Multiple foci of spongiosis were observed in the molecular layer neuropil. Purkinje neurons with accumulation of intraneuronal empty vacuoles of different size were also apparent. Some neurons in the cerebellar, general somatic efferent oculomotor, and red nuclei were pale and were affected by diffuse gliosis. The remainder of the brain was histologically normal. Based on these findings, a definitive diagnosis of granuloprival CCD was made.

### 2.2 Case 2

**FIGURE 3:**
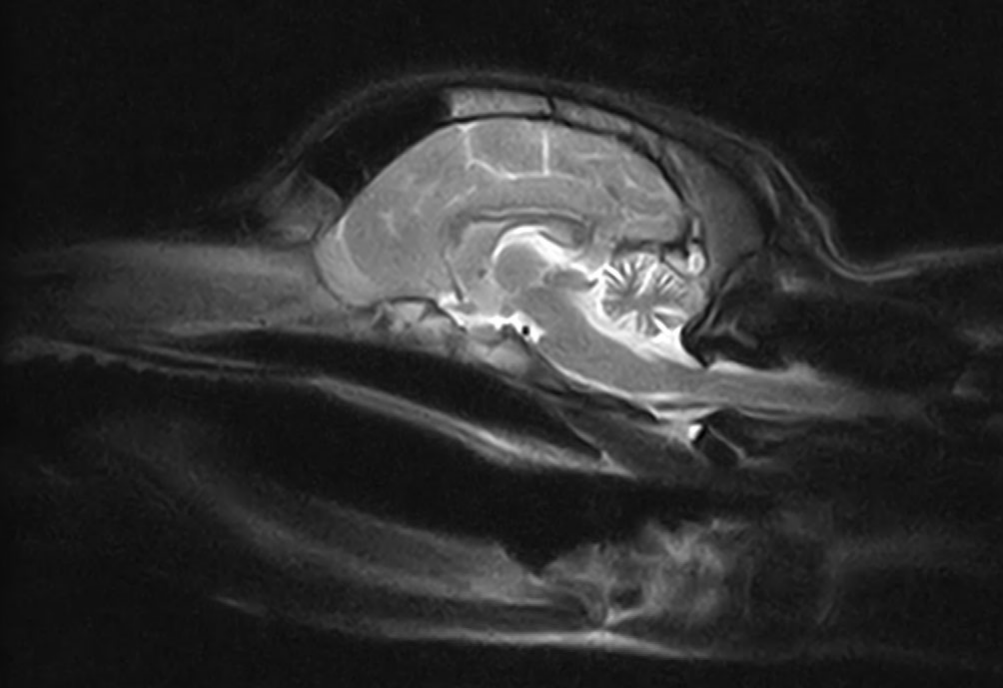
T2‐Weighted midline sagittal magnetic resonance image of the brain for Case 2.

**FIGURE 4:**
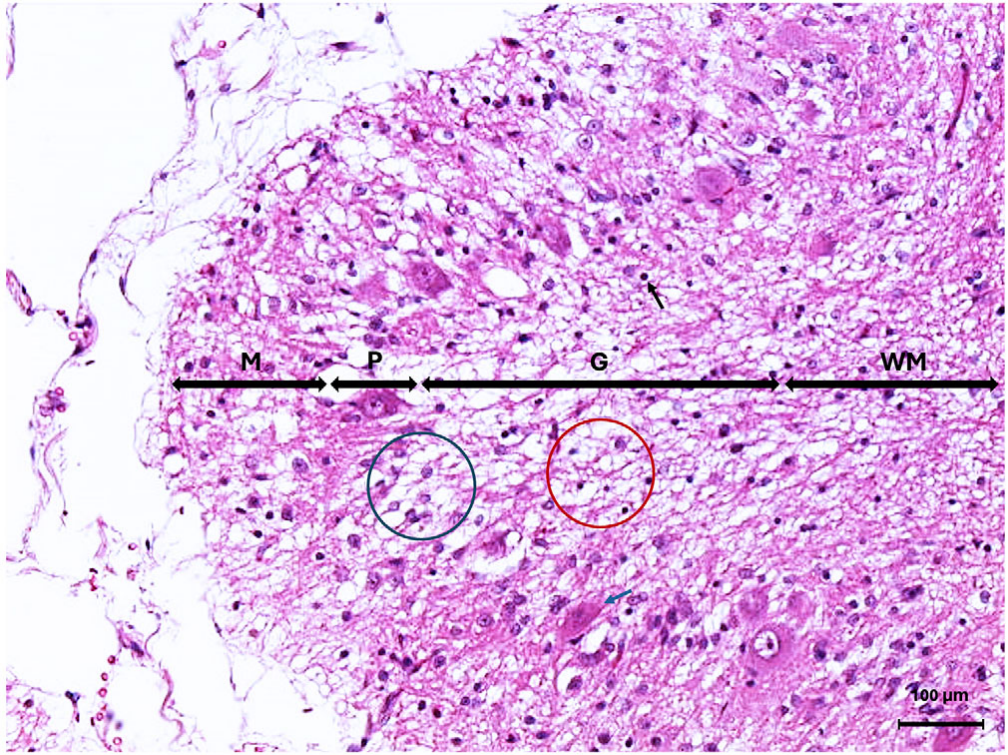
Microscopic image of the cerebellar cortex of Case 2 stained with Hematoxylin and Eosin. The granular cell layer (G) is displaying marked hypocellularity of the granule cell neurons with marked vacuolation of the neuropil (red circle), as well as shrunken pyknotic granular cell neurons (black arrow). There is rare loss of Purkinje neurons (blue circle) as well as rare shrunken angular hypereosinophilic Purkinje neurons (blue arrow) in the Purkinje cell layer (P). The molecular layer (M) size is reduced. White matter (WM).

A 7‐month‐old intact male Lagotto Romagnolo dog was presented with a 4‐month history of progressive cerebellar ataxia involving all limbs as well as intention tremors of the head and neck. Eye movements were reported to be normal. All 5 littermates of the puppy were reported to be normal. General examination did not identify any abnormality. Neurological examination disclosed a wide‐based stance as well as cerebellar ataxia characterized by hypermetria involving all limbs, intention tremors of the head and neck, and bilaterally absent menace response despite normal vision. The remainder of the neurological examination was normal. Neuroanatomical localization was to the cerebellum. Differential diagnoses included CCD, intracellular vacuolar storage disease, cerebellar hypoplasia, neosporosis, or a congenital malformation of the cerebellum such as Dandy Walker malformation, as well as a hypomyelination disorder or neuroaxonal dystrophy. Serum biochemistry and CBC were normal. Genetic testing for the autophagy‐related ATG4D gene mutation associated with intracellular vacuolar storage disease in Lagotto Romagnolo dogs identified homozygous copies of the normal allele, ruling out the disease. Serology for *Toxoplasma gondii* and *Neospora caninum* was negative. Magnetic resonance imaging (Vet‐MR Grande 0.25 Tesla, Esaote, Genova, Italy) of the head was performed using a head coil (DPA Head Coil, Esaote, Genova, Italy), under general anesthesia. Magnetic resonance imaging disclosed marked diffuse decreased size of all lobes and lobules of the cerebellar vermis and hemispheres with marked widening of all cerebellar fissures, most obvious at the primary fissure, containing fluid isointense to cerebrospinal fluid on T1‐Weighted, T2‐Weighted, and fluid attenuation inversion recovery sequences (Figure 3). No contrast enhancement or transforaminal herniation was observed. The brainstem‐to‐cerebellum area ratio was 132% (normal, <96%) indicative of decreased cerebellum size (Figure 3).^12^ Cerebrospinal fluid was collected from the cerebellomedullary cistern under general anesthesia and analysis, including total nucleated cell count and protein concentration, was normal. Polymerase chain reaction testing on cerebrospinal fluid for canine distemper virus, Bartonella species, *Cryptococcuss species*, *Borrelia burgdorferi*, *Neospora caninum*, and *Toxoplasma gondii* was negative. Based on the history of progressive clinical signs, decreased cerebellar size on magnetic resonance imaging, negative infectious disease and negative genetic testing for the autophagy‐related ATG4D gene mutation, the suspected diagnosis was therefore that of CCD although the possibility of cerebellar hypoplasia could not be fully ruled out. The dog's clinical signs remained stable over the next 9 months until rapid deterioration of clinical signs was noticed, rendering the dog unable to walk. At this time, the owners elected for euthanasia given the compromised quality of life and consent for necropsy was given. On necropsy, all lobes and lobules of the cerebellar vermis and hemispheres were markedly and diffusely decreased in size with marked thinning of the cortex upon sectioning. Microscopically, the cerebellar folia were markedly flattened. Marked diffuse thinning and hypocellularity of the granular cell layer with marked loss of granular cells was observed, leading to almost complete absence of this layer with the presence of marked vacuolation of the neuropil (Figure 4). A few remaining granular cells were swollen and vacuolated or shrunken with pyknotic nuclei. Glial cells mainly in the form of astrocytes were diffusely present in this layer. The molecular layer was also mildly decreased in thickness, but otherwise unremarkable. The Purkinje cell layer appeared unaffected other than for rare loss of Purkinje cells. Multifocally, a few scattered Purkinje cells exhibited either shrunken and angular, hypereosinophilic cytoplasm and pyknotic nuclei or rarely swollen nuclei with central chromatolysis. A moderate increase in glial cells was diffusely observed in the white matter of the mesencephalon. The remainder of the brain was histologically normal. Based on these findings, a definitive diagnosis of granuloprival CCD was made.

## 3 DISCUSSION

Granuloprival CCD is a relatively uncommon form of CCD and the exact etiology remains to be elucidated and might be multifactorial.^1^, ^5^, ^9^, ^10^, ^11^ Canine herpesvirus has been reported to cause granular cell depletion with associated Purkinje neuron loss along with foci of infiltrating mononuclear cells and malacia.^13^ Intrauterine or perinatal infection with feline parvovirus can cause hypoplasia of the granular cell layer in cats, and canine parvovirus has been detected in dogs with cerebellar hypoplasia, but not in CCD cases.^14^, ^15^ Alternatively, an immune‐mediated etiology for granuloprival CCD is suspected in the Coton de Tuléar breed given the presence of marked diffuse T cell infiltration of the granular cell layer.^5^ In our 2 cases, histopathological findings were not consistent with an infectious or immune‐mediated etiology given the lack of inflammatory cell infiltration or perivascular cuffing, making an infectious or immune‐mediated etiology unlikely. Furthermore, infectious disease testing was negative. Given the lack of immune‐mediated or infectious histopathologic changes in the majority of granuloprival CCD cases, a genetic basis is suspected even when individual puppies are affected.^2^, ^3^, ^4^, ^6^, ^7^, ^8^, ^9^, ^10^, ^11^, ^12^ Similarly, a degenerative etiology most likely of genetic origin is suspected in our 2 cases because of exclusion of infectious and inflammatory causes as well as the onset of clinical signs in the first few weeks of life and progressive worsening making anomalous, neoplastic, traumatic, and vascular causes unlikely. However, a metabolic or toxic etiology cannot by fully ruled out. An autosomal recessive mode of inheritance could explain the occurrence of single affected puppies in our 2 cases, should a genetic basis for the granuloprival degeneration be identified.^6^

Similar to domestic species, most forms of CCD in humans primarily affect the Purkinje neurons with secondary retrograde degeneration of the granule cell neuron layer.^16^ However, a rare subtype of CCD in humans termed Norman type shares similarities with the granuloprival degeneration observed in our 2 cases in dogs.^17^ Norman type CCD is also characterized by primary degeneration of the granule cell neuron layer of the cerebellum with sparing of the Purkinje neuron layer, and also has a juvenile onset.^17^, ^18^ A genetic basis for Norman type CCD is suspected given its familial occurrence.^18^

The mechanisms underlying a genetically‐mediated selective loss of granule cell neurons also remain unclear.^6^, ^9^, ^10^, ^11^ A channelopathy resulting in excitotoxicity and apoptosis of granule cell neurons as occurs in homozygous weaver mice has been postulated in dogs.^6^, ^19^, ^20^ In the former, however, granular cell neurons undergo premature degeneration in the external granular layer before migration whereas in our 2 cases the few remaining granule cell neurons were located in the granule cell neuronal layer indicating appropriate migration.^20^ Genetic targets capable of causing degeneration of granular cell neurons without affecting their prior migration are therefore considered more plausible.^9^ These include (a) the sonic hedgehog mitogen that interacts with a set of receptor molecules on granule cell neuron precursors delivering essential proliferative signals from Purkinje cells; (b) the bone morphogenetic protein signaling cascade that intermittently triggers all stages of proliferation as well as the postmitotic life of granule cell neurons; and (c) the functional ɑ‐amino‐3‐hydroxy‐5‐methyl‐4‐isoxazolepropionic acid receptors in cerebellar granule cell neurons as is seen in waggler, stargazer and stargazer 3 Jackson mice.^21^, ^22^, ^23^, ^24^

Antemortem presumptive diagnosis of CCD involves ruling out anomalous, neoplastic, inflammatory (immune‐mediated and infectious), and metabolic causes as well as other degenerative causes.^1^ Magnetic resonance imaging can be normal, usually early in the disease process, or can disclose a decreased cerebellar size with decreased thickness of the cerebellar folia, resulting in widening of the associated cerebellar fissures with cerebrospinal fluid.^1^, ^8^, ^11^ Clinically, antemortem differentiation of CCD from cerebellar hypoplasia relies on criteria such as onset and progression of clinical signs.^25^ In cerebellar hypoplasia, onset of clinical signs is typically from birth with stabilization of clinical signs thereafter or possible slight improvement because of learned compensatory mechanisms. Whereas in CCD onset is often from a few weeks, months, or rarely years after birth, with progressive worsening of clinical signs over time.^25^ However, neonatal forms of CCD are reported such as in the Beagle, and a transient plateau of clinical signs can be seen with CCD as reported in Case 2, complicating antemortem clinical differentiation of CCD and cerebellar hypoplasia.^26^ Hence definitive diagnosis of CCD requires histopathology.^1^

The histological changes observed in both cases correlate well with the functional relationship of the granule cell neuronal layer. Atrophy of the molecular layer observed in both cases is explained by the loss of the parallel fibers.^25^ Conversely, loss of granule cell neurons did not cause substantial anterograde degeneration of Purkinje neurons because of sufficient collateral stimulatory input from the olivary nucleus, which did not have any pathological changes.^25^ The intraneuronal accumulation of eosinophilic granular material observed in Purkinje cells could reflect their abnormal metabolism because of loss of synaptic contact with granular cell neurons. Although pathological changes were found in the neurons of the cerebellar, general somatic efferent oculomotor, and red nuclei, no associated clinical signs were observed.

Case 1 had a juvenile onset of clinical signs with slow progression. Case 2 had a juvenile onset of clinical signs with an initial slow progression, followed by a plateau for 9 months, and then rapid deterioration. Age of onset of clinical signs in Case 2 is similar to the only other granuloprival CCD case reported in the Lagotto Romagnolo breed, but progression differed in that clinical signs progressed rapidly in the previously reported case.^8^ Cerebellar cortical degeneration with primary involvement of the Purkinje neurons, as well as neuronal heterotopia involving the cerebrum, pons, and cerebellum, also have been reported as single cases in the Lagotto Romagnolo breed, and it remains uncertain whether these represent the spectrum of a single disease involving neuronal migration or neuronal development and survival, or both.^8^, ^27^

Because CCD is a degenerative condition with no known treatment, clinical signs progress until quality of life becomes compromised at which time euthanasia usually is performed.^1^ All dogs with granuloprival CCD are reported to have been euthanized between 4 weeks and 6 months after the onset of clinical signs owing to compromised quality of life.^2^, ^3^, ^4^, ^5^, ^6^, ^7^, ^8^, ^9^, ^10^, ^11^ In Case 1, euthanasia was performed 6 weeks after the onset of clinical signs, but in Case 2, euthanasia was performed 13 months after the onset of clinical signs, representing the longest reported survival time.

## 4 CONCLUSION

In conclusion, we respectively report the first and second histopathologically confirmed cases of granuloprival CCD in the Yorkshire Terrier and Lagotto Romagnolo breeds. Granuloprival CCD therefore should be considered as a differential diagnosis in Yorkshire Terrier and Lagotto Romagnolo dogs with post‐natal progressive clinical signs of cerebellar dysfunction. Additional studies are required to determine the etiology of granuloprival CCD in these breeds.

## CONFLICT OF INTEREST DECLARATION

Authors declare no conflict of interest.

## OFF‐LABEL ANTIMICROBIAL DECLARATION

Authors declare no off‐label use of antimicrobials.

## INSTITUTIONAL ANIMAL CARE AND USE COMMITTEE (IACUC) OR OTHER APPROVAL DECLARATION

Authors declare no IACUC or other approval was needed.

## HUMAN ETHICS APPROVAL DECLARATION

Authors declare human ethics approval was not needed for this study.
